# The strategies to cure cancer patients by eradicating cancer stem-like cells

**DOI:** 10.1186/s12943-023-01867-y

**Published:** 2023-10-18

**Authors:** Yansui Mai, Jiyan Su, Chuan Yang, Chenglai Xia, Liwu Fu

**Affiliations:** 1 Affiliated Foshan Maternity and Child Healthcare Hospital, Southern Medical University, Foshan, China; School of Pharmaceutical Sciences, Southern Medical University, Guangzhou, China; 2 State Key Laboratory of Oncology in South China; Collaborative Innovation Center for Cancer Medicine; Guangdong Esophageal Cancer Institute; Sun Yat-sen University Cancer Center, Guangzhou, 510060 China

**Keywords:** Cancer stem cells, Tumor microenvironment, Immune evasion, Targeted therapies

## Abstract

Cancer stem-like cells (CSCs), a subpopulation of cancer cells, possess remarkable capability in proliferation, self-renewal, and differentiation. Their presence is recognized as a crucial factor contributing to tumor progression and metastasis. CSCs have garnered significant attention as a therapeutic focus and an etiologic root of treatment-resistant cells. Increasing evidence indicated that specific biomarkers, aberrant activated pathways, immunosuppressive tumor microenvironment (TME), and immunoevasion are considered the culprits in the occurrence of CSCs and the maintenance of CSCs properties including multi-directional differentiation. Targeting CSC biomarkers, stemness-associated pathways, TME, immunoevasion and inducing CSCs differentiation improve CSCs eradication and, therefore, cancer treatment. This review comprehensively summarized these targeted therapies, along with their current status in clinical trials. By exploring and implementing strategies aimed at eradicating CSCs, researchers aim to improve cancer treatment outcomes and overcome the challenges posed by CSC-mediated therapy resistance.

## Introduction

CSCs were firstly identified in acute myeloid leukemia (AML), where they exhibited stem cell-like and cancer cell-like properties and were found to be the sole cause of the initiation and progression of the corresponding cancer [1]. The tumorigenicity and self-renewal ability are indispensable properties of CSCs. To identify potential populations of CSCs, an important test was developed to determine the ability of CSCs to form tumors at low cell densities. This test, known as Extreme Limiting Dilution Assays (ELDA) became widely used as a gold standard to estimate active CSCs frequencies [2]. Studies have shown that as few as 100 cells exhibiting the CSCs phenotype were capable of forming tumors in mice, while tens of thousands of cells with an alternative phenotype were unable to do so [3]. Furthermore, CSCs must have the ability to sustain themselves and continue to generate cells with the same tumorigenicity and primitive tumor-forming capabilities [4]. A study provided evidence by using human cells with CD45 marker from the bone marrow of AML transplant recipients. These cells were found to have the same capacity to induce most subtypes of AML in secondary recipients, highlighting the self-renewal property of CSCs [5]. Unfortunately, CSCs are highly resistant to systemic anti-cancer therapies due to their complicated drug resistance mechanisms and active DNA repair capacity. Even after successfully resecting the primary tumor, the dormant disseminated CSCs can contribute tumor relapse and metastasis due to their long-term capacity for self-renewal [6].

Based on CSC theory, only a small subset of cells sustain tumorigenesis and contribute to cellular heterogeneity in primary tumors. These CSCs share certain properties of stem cells although they are not necessarily derived from stem cells found in normal tissues [7]. This suggests CSCs are a particular cell state that could initiate tumor growth. One widely accepted hypothesis is that cells can be transformed from more specialized, non-stem cells into stem-like cells through a process called epithelial-mesenchymal transition (EMT) [8]. During EMT, cancer cells undergo heritable phenotypic changes that are brought about by epigenetic modifications, rather than the introduction of new genetic alterations. As a result, the cancer cells lose their epithelial characteristics, such as cell–cell junctions and apical-basal polarity and gain mesenchymal features such as elongated, fibroblast-like morphology. These EMT-activated cancer cells exhibit CSC-like characteristics, including the expression of cell markers associated with stemness, as well as the ability to form tumors [9, 10]. The coexistence of both epithelial and mesenchymal characteristics allows cancer cells to survive, metastasize and colonize distal organs. Current research on CSCs focuses on understanding how these cells interact with the surrounding TME. This interaction involves various bidirectional cellular mechanisms, such as direct cell-to-cell contact, ligand-receptor interactions, and interactions with non-tumor cells that are present in the TME [11]. These interactions play a role in driving tumor progression. The chronic inflammatory and immunosuppressive TME is thought to be the primary factor contributing to EMT and the high stemness of CSCs [12]. Signals from the TME activate intracellular signaling pathways, leading to changes in biomarker expression, and promote immune evasion, which ultimately converge to maintain CSCs properties [13, 14].

Evidence suggests that conventional cancer therapies often fail to completely eliminate cancer cells that have undergone a switch to the CSC state. This switch is made possible through the activation of the EMT program, which can lead to CSC-related clinical recurrence. In light of these findings, eradicating or differentiating the CSC subpopulation appears to be a potential strategy for cancer treatment. However, despite these promising prospects, there is still a long way to go in comprehensively demonstrating the formation and development of CSCs, as well as in developing targeted therapies to counter them. This review focuses on current status of research and development in eradicating CSCs. The strategies discussed include cell biomarker-/pathway-targeting strategies, TME-targeting strategies, immune modification strategies, and agent-induced differentiation strategies. By examining these different approaches, this review aims to provide insight into the progress made thus far in the field of CSC-targeted therapies and to highlight the potential for future advancements in this area.

## CSC biomarkers, stemness-associated pathways targeting therapies

Cell markers that distinguish CSCs from normal cells have potential applications in the diagnosis, treatment, and prognosis of cancer.

### CSCs biomarkers

**Fig. 1:**
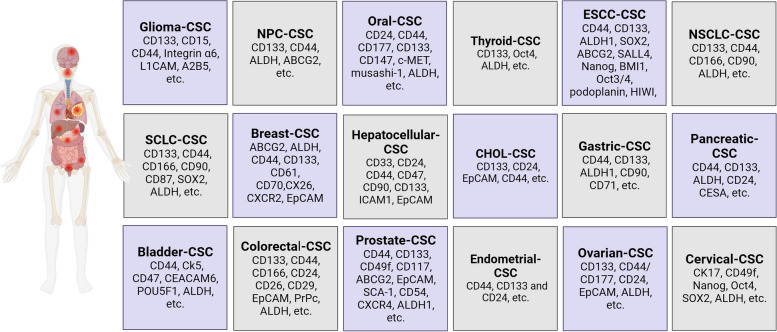
Specific biomarkers for CSCs in different types of cancer

Different biomarkers have been utilized to identify CSCs, with cancer type-specific biomarkers being well-reviewed and showed in Fig. 1 [15–33]. This revealed common biomarkers for CSCs including CD44, CD133, aldehyde dehydrogenase (ALDH), and epithelial cell adhesion molecule (EpCAM).

CD44 expression is upregulated in cancer cell subpopulations and serves as a molecular marker of CSCs. CD44^+^ cells isolated from cancer patients were capable of initiating tumor growth when transplanted into immunocompromised mice and showed increased resistance to radiochemotherapy [34]. CD44 is a crucial receptor for hyaluronic acid (HA) and extracellular matrix (ECM). Binding of HA or osteopontin to CD44 results in the activation of the STAT3, Oct4-Sox2-Nanog or c-Src kinase signaling pathways, which are known to promote anti-apoptosis and chemoresistance in CSCs [35]. In addition, CD44 also acts as a receptor for growth factors and cytokines such as transforming growth factor (TGF)-β, vascular endothelial growth factor (VEGF), and matrix metalloproteinase (MMP). Through these interactions, CD44 facilitates intercellular communication and mediates process such as EMT. This suggests that CD44 plays a key role in coordinating the cellular responses to various environmental signals to contribute to the self-renewal and invasiveness of CSCs [36]. Clinical studies have highlighted the clinicopathological role of CD44 in promoting tumorigenesis and have identified CD44 as a potential therapeutic target.

CD133, a pentosan membrane glycoprotein, is one of the most well-characterized biomarkers used for isolation of CSCs. It is found to promote tumorigenicity, spheroid formation ability, and the EMT program [37]. One significant finding is that cancer cells from patients with lung cancer that express CD133 could be maintained in specific media indefinitely. In contrast, cancer cells lacking CD133 died within a few weeks of culture [38]. Moreover, the CD133^+^ population consistently generated tumors in immunocompromised mice, but not CD133^−^ cells [38]. These observations indicate the crucial role of CD133 in the maintenance and growth of cancer cells. Additionally, CD133 has been found to recruit histone deacetylase 6 (HDAC6) to deacetylate β-catenin, thereby causing the stabilization and nuclear localization of β-catenin. This, in turn, promotes the interaction between β-catenin and T-cell factors (TCF), resulting in the acceleration of cancer cell growth [39]. CD133 also interacted with p58 to activate the PI3K/Akt pathway, which further regulated CSC proliferation and tumorigenesis [40]. The combined expression of CD133 and CD44 in colorectal cancer has been shown a sevenfold increase in tumorigenicity. However, CD133 alone showed a change of more than 1.45 times, and CD44 alone led to a twofold increase in tumorigenicity [41]. Overall, these findings highlight the critical role of CD133 in the maintenance, growth, and tumorigenicity of cancer cells.

The EpCAM is considered as a multi-functional transmembrane protein associated with the regulation of cell adhesion, proliferation, migration, stemness, and EMT of cancer cells [42]. It served as an oncogenic signal transducer that is more accessible to be targeted by antibodies than those on normal cells [43]. The expression of EpCAM has been found to be tumorigenic, meaning that it is involved in the formation and growth of tumors. Transplantation of EpCAM^+^CD45^−^ cells isolated from patients with hepatocellular carcinoma (HCC) into NOD/SCID mice triggered tumor formation, while EpCAM^−^CD45^−^ cells did not have the same effect [44]. EpCAM controls cell cycle progression and differentiation via regulated intramembrane proteolysis (RIP). Following RIP-mediated EpCAM cleavage, the EpCAM intracellular domain (EpICD) is released and translocated into nucleus, where it binds to transcription factors and adaptor molecules such as four-and-a-half LIM domains protein 2 (FHL2), β-catenin, and TCF to initiate the stemness-related gene expression and cell reprogramming [45]. The binding of TCF/β-catenin complex, in turn, could bind to EpCAM promoter and regulate EpCAM gene expression [46]. The interaction of EpCAM and β-catenin signaling creates a positive feedback loop to promote the enrichment of the EpCAM^+^ cell population, which contributes to the promotion of cell self-renewal, differentiation, and invasiveness [47]. In addition, the overexpression of EpCAM has been found to promote EMT and upregulate cellular transcriptional factors such as Nanog, Oct4 and SOX2 [48]. The high expression of EpCAM indicates poor differentiation grade that directly associated with poor survival rate [49]. The above findings suggest that the measurement of EpCAM expression could serve as a prognostic marker for cancer patients and its antibody-targeted therapy could have potential application.

ALDHs are essential regulators of aldehyde metabolism in human body that protect organisms from active aldehydes-induced damage. ALDHs deficiency and polymorphisms in organisms are associated with diseases such as Parkinson’s disease, Type 2 hyperprolinaemia, hypertension and Sjögren-Larsson syndrome [50], while upregulated ALDH expression indicates a high degree of tumor malignancy [51, 52]. However, the exact mechanism of the effect by which ALDHs contribute to the maintenance of CSCs has not been clarified. Current research suggests that the interactions of ALDHs with retinoic acid (RA), reactive oxygen species (ROS), and reactive aldehydes may contribute to their functional roles [50]. ALDHs suppress the transformation of retinal to RA, which in turn reduce the ability of RA to inhibit clonogenic and tumorigenic potential [53]. The administration of all-trans RA (ATRA) inhibited the expression of ALDH1 and the activation of nuclear factor erythroid 2-like 2 (NRF2), resulting in the attenuation of CSC-like properties [54]. In addition, ALDHs regulate the expression of hypoxia-inducible transcription factors 2 (TIF-2) and decrease the ROS accumulation in tumor, thereby preventing CSCs apoptosis [55]. ALDHs can detoxify and metabolize cytotoxic chemotherapeutic drugs by oxidizing the aldehyde groups of chemotherapeutic drugs into non-toxic carboxylic acids. This is the main cause for CSCs maintaining drug resistance [56]. On the other hand, ALDH is associated with Nanog expression. It’s reported that the activity of ALDH is regulated by Nanog through the Notch1/Akt signaling pathway to induce CSC stemness and cellular radioresistance [57]. SOX2/Oct4 interacts with the Wnt/β-catenin signaling pathway to maintain CSC stemness and therapy resistance [58, 59], and as an upstream regulator of Nanog, SOX2/Oct4 is thought to be involved in modulating ALDH activity to control CSC properties. Suppression of ALDH activity can induce apoptosis and alleviate drug resistance [60].

Although the number of CSC markers identified is growing, it has been reported that not all of these markers are equally effective in identifying CSCs. On this case, investigation that focuses on stemness-associated CSCs signaling networks is necessary to comprehensively understand the role of CSCs in tumor progression.

### Stemness-associated pathways

**Fig. 2:**
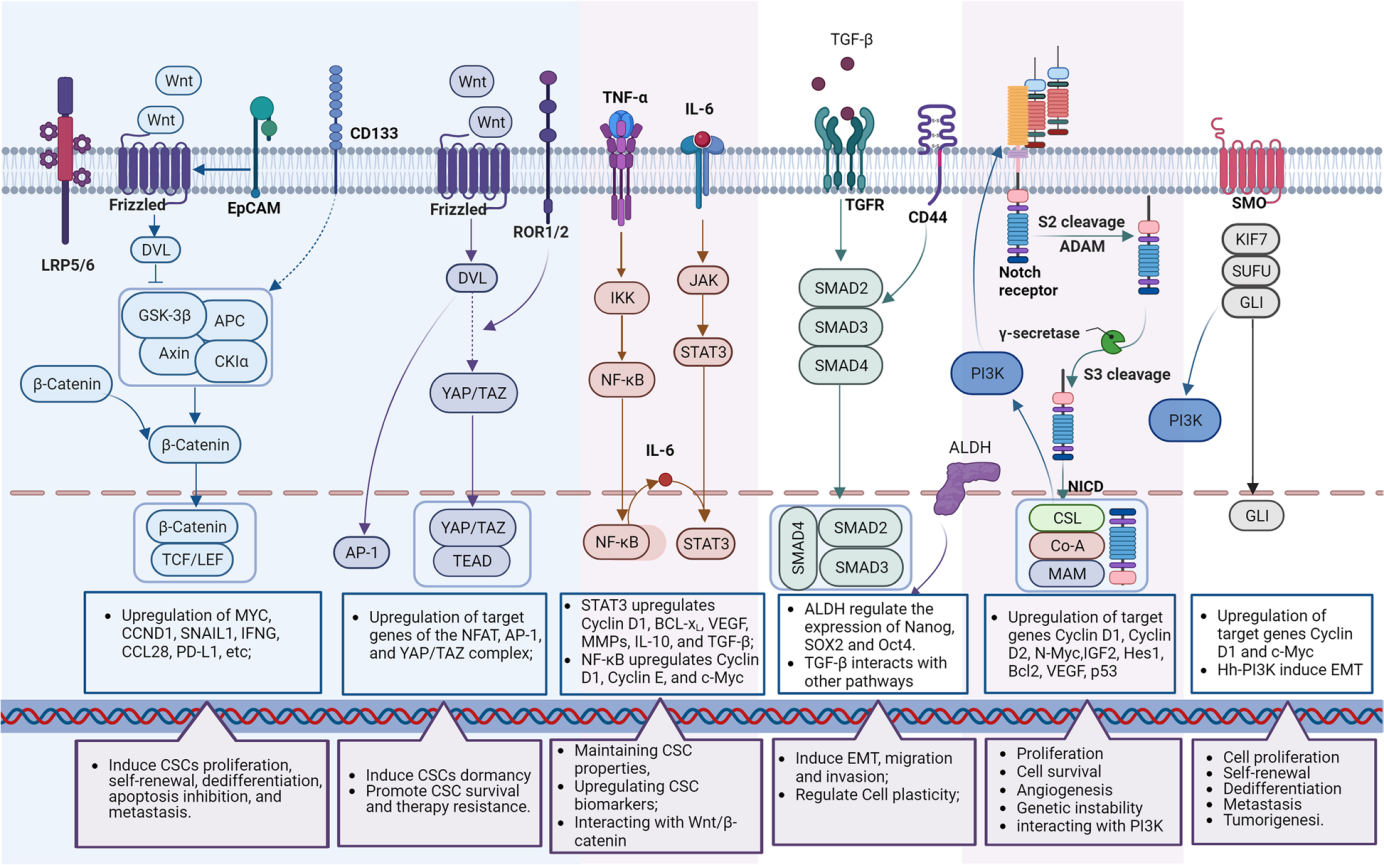
Contribution of aberrantly activated pathways in CSCs. Dysregulated pathways activate the transcriptional activity of target genes, thereby contributing to the maintenance of the proliferation/dormancy, survival, self-renewal, migration, and evasion properties of CSCs

Deregulated signaling pathways controlling self-renewal and differentiation of CSCs are identified during CSC-induced tumor initiation, including Wnt/β-catenin, Notch, Hedgehog (Hh), and Hippo signaling pathways. In addition, these self-renewal related pathways interact with other oncogenic signaling pathways, including the nuclear factor-κB (NF-κB), signal transducer and activator of transcription 3 (STAT3), and PI3K/Akt signaling pathways, to inhibit apoptosis and promote cell proliferation [61] **(**Fig. 2**)**.

#### Wnt signaling pathway

The canonical Wnt signaling pathway, also known as Wnt/β-catenin signaling, is activated when Wnt ligands bind to Frizzled (FZD) family receptors and LRP5/6 coreceptors. This binding then transduces signals into the β-catenin signaling cascade. In the absence of canonical Wnt ligands, the β-catenin complexes are phosphorylated and subsequently degraded in the cytoplasm [62]. However, in the presence of Wnt ligands, the binding of Wnt ligands and receptors induces the accumulation of β-catenin in cytoplasm, which is then phosphorylated by glycogen synthase kinase 3β (GSK3β). The phosphorylated β-catenin translocates to the nucleus to activate the transcription of CCND1 and MYC directly, followed by upregulation of SNAIL1, IFNG, CCL28, PD-L1, and others, to initiate the EMT process and regulate cell progression and differentiation [63]. Canonical Wnt signaling is also reported to interact with canonical TGF-β pathway to induce the development of EMT [64]. The accumulated β-catenin in the nucleus reinforces canonical Wnt signaling in response to TGF-β reactions [65]. Both nuclear β-catenin and TGF-β repress the expression of E-cadherin gene. Loss of E-cadherin expression during EMT is believed to promote metastasis by allowing cancer cells to dissociate and invade [66, 67].

In contrast, the non-canonical Wnt signaling cascade induces CSC dormancy [68]. In the process of Wnt/PCP signal transduction, ROR1 receptors induce RhoA-mediated YAP stabilization and nuclear translocation, reprogramming LGR5 + proliferating CSCs into LGR5-dormant CSCs [69]. The ROR2 receptor induces CSC dormancy by inhibiting canonical Wnt signaling especially through promoting the degradation of β-catenin [70]. It is worth noting that proliferative CSCs are more vulnerable to DNA damage caused by chemotherapy, while non-proliferative CSCs are more resistant to such treatments [71]. Both canonical and non-canonical Wnt signaling pathways contribute to therapeutic resistance and subsequent relapse occurs due to the expansion of proliferative CSCs and the survival of dormant CSCs.

#### Notch signaling pathway

The canonical Notch cascade operates by direct cell-to-cell contact. This contact is initiated when transmembrane ligands expressed by signal-sending cells ligate receptors expressed on signal-receiving cells [72]. The ligand-mediated activation induces a series of proteolytic cleavages in Notch family receptors and releases the receptors’ NICD [73]. The NICD then translocates to the nucleus to initiate the Notch target genes transcription [74].

It is important to note that abnormal Notch signaling has been found to facilitate self-renewal and metastasis of CSCs [75]. Additionally, there is a crosstalk between Notch signaling and other pathways, such as TGF-β and EGFR. Activated Notch pathway leads to an expansion of TGF-β-induced smad2/3 signaling and EGFR inhibitors resistance [14, 76]. Notably, the pathogenic role of the Notch pathway appears to be tumor type dependent. Aberrant activation of Notch signaling promotes the development of most solid tumors but suppresses myeloid malignancies [77, 78]. Therefore, a comprehensive demonstration of the mechanisms of Notch signaling in specific cancers is essential for the successful development of therapeutics targeting this pathway.

#### Hh signaling pathway

Accumulating evidence suggests that aberrant Hh activation leads to tumor transformation, progression, and therapeutic resistance in a variety of cancers. The Hh signaling drives cancer progression by regulating cancer cell proliferation, malignant transformation, metastasis, and the CSC expansion [79]. Upon binding of the Hh ligands, such as Sonic (SHh), India (IHh), or Desert (DHh) to their receptors, the Hh signaling pathway is activated to initiate GLI transcription factors. The nucleus localization of GLI drives the expression of Hh target genes, most of which are associated with proliferation, cell survival, angiogenesis, and genetic instability [80]. The relatively high expression of GLI1, GLI2, PTCH1, and Hedgehog interacting protein (Hip) in CSCs indicates that the Hh signaling is preferentially activated in this cellular compartment [81].

The Hh signaling pathway has been shown to crosstalk with other oncogenic pathways in many cancer types, such as RAS/RAF/MEK/ERK, PI3K/Akt, EGFR, and Notch [82]. KRAS has been reported to activate Hh signaling by modulating the expression, phosphorylation, and degradation of GLI1. Simultaneous activation of RAS/RAF/MEK/ERK and Hh pathway increases the proliferation of CSCs and their potential for tumor formation. Activation of the PI3K/Akt pathway promotes GLI1 phosphorylation and nuclear translocation, which in turn increases the expression of GLI1 target genes and its oncogenic function. EGFR cooperates with Hh signaling to enhance CSCs attributes. The combined activation of EGFR and Hh pathway synergistically promote tumor formation, in part due to EGFR-GLI regulated RAF/MEK/ERK target JUN/AP-1 expression. JUN/AP-1 interacts with GLI protein to activate downstream GLI/EGF target genes.

#### Hippo signaling pathway

Hippo pathway is a conserved signaling that modulates cell proliferation, differentiation, and survival. The dysregulation of the Hippo pathway can cause a variety of diseases including cancer [83]. The Hippo pathway status controls the dynamic localization of YAP/TAZ between nucleus and cytoplasm. Once activated, the Hippo pathway limits cell proliferation through phosphorylation and degradation of YAP/TAZ in cytoplasm. In contract, when the Hippo pathway is off, YAP/TAZ translocates into nucleus and binds with TEAD to induce cell proliferation, survival and migration [84]. As highly tumor-associated transcriptional regulators, YAP/TAZ are pervasively activated in human malignancies and initiates the growth of most solid tumors [85]. In addition, Hippo signaling and subsequent activation of YAP/TAZ is a major mechanism involved in CSC therapy resistance and therefore a potential target for CSC eradication [86].

#### Interactions between self-renewal pathways and oncogenic pathways

Self-renewal signaling pathways contribute to cell proliferation, survival, and differentiation properties in CSCs. In the meanwhile, oncogenic pathways including NF-κB, STAT3, and PI3K/Ak signaling pathways, participate cell stemness regulation by controlling their downstream gene expression, such as cytokines, growth factors, apoptosis genes in CSCs [61].

The NF-κB signaling pathway (including canonical and alternative NF-κB signaling) plays a crucial role in the regulation of inflammation and immune system, and its overactivation is associated with cancer progression by promoting cell proliferation, survival, angiogenesis and invasion [87]. Researches demonstrated that both canonical and alternative NF-κB and their targeted genes are upregulated in the majority cancers [88, 89]. The increased transcriptional activity of NF-κB-targeted genes leads to the upregulated expression of Cyclin D1, Cyclin E, and c-Myc, which are responsible for cell proliferation [90]. What’s more, NF-κB participates in the regulation of a variety of carcinogenic mechanisms, including enhancing inflammatory responses through secretion of tumor necrosis factor (TNF)-α, IL-1β, IL-6, MCP1, COX2, and iNOS; promotion of EMT by the expression of vimentin and Twist; remodeling the extracellular matrix through induction of angiogenesis factors (e.g., IL-8 and VEGF); and facilitating invasion and metastasis through MMPs [91].

STAT3 signaling has been shown to have central role in CSCs in promoting cell proliferation, survival, tumor invasion, angiogenesis, and immunosuppression [92]. IL-6 acts directly on cancer cells to induce the expression of STAT3 target genes, including Cyclin D1, BCL2-like protein 1 (BCL-x_L_), VEGF, MMPs, IL-10, and TGF-β, thereby contributing to the maintenance of CSC properties [93]. Activated STAT3 is essential for maintaining the expression of CSC biomarkers such as CD24, CD34, CD38,CD44, CD90, CD133, and ALDH [94]. Additionally, induction of EMT and expansion of the CSC population were observed followed by STAT3 activation [95]. NF-κB and STAT3 are major factors regulating CSC angiogenesis and invasiveness during cancer progression, and their activation and interaction play an important role in controlling communication between cancer cells and inflammatory cells [96]. NF-κB is a key transcription factor that drives the expression of IL-6 [97]. In particular, NF-κB is overexpressed in multiple human cancers and activated STAT3 in tumor also induce IL-6. This creates a positive-feedback loop and suggests that IL-6 may be the basis for NF-κB and STAT3 interaction [98]. Wnt/β-catenin is involved in the IL-6 feedback loop to provides an important basis for inflammation-induced tumorigenesis [99]. The Wnt ligands are upregulated in inflammatory tissues and produce IL-1β and IL-6 by acting on the transcriptional factors NF-κB and STAT3. These cytokines activate STAT3, which facilitates Wnt ligands production and to Wnt/β-catenin pathway activation [68].

PI3K/Akt pathway is a highly conserved major transduction network in cells that promotes cell survival, growth and proliferation [100]. Dysregulation of PI3K/Akt pathway is achieved by variety of mechanisms, including loss or inactivation of the tumor suppressor PTEN, mutation or amplification of PI3K, and activation of tyrosine kinase growth factor receptors or oncogenes upstream of PI3K [101]. Increasing evidence indicated that activation of PI3K in cancers is associated with cancer progression by enhancing CSC phenotype, EMT, and therapy resistance [102]. Downregulation of PTEN induces PI3K activation to promote cell survival, maintenance of stemness, and tumorigenicity of prostate cancer stem-like cell population [103]. Indeed, important crosstalk and interactions between Notch signaling and PI3K/Akt pathway has been observed. Overactivation of PI3K/Akt pathway can upregulate the expression of Notch 1 ligand via NF-κB. Notch 1 in turn supports PI3K/Akt activity and prevents their dephosphorylation by inhibiting protein phosphatase 2 (PP2A) and PTEN activation, thereby supporting and promoting cancer progression [104]. Additionally, membrane-tethered Notch may activate the PI3K/Akt pathway to promote the transcription of IL-10 and IL-12 [105]. The cross talk between Hh and PI3K/Akt is a crucial regulator of EMT, Hh-GLI induces EMT and invasion and metastasis by activation of PI3K/Akt [106]. Furthermore, the mechanistic Target of Rapamycin (mTOR) is a downstream component of the KI3K/Akt pathway and has been reported to be involved in programmed cell death protein 1 (PD-1) expression through Hh signaling cascade, independent of SMO [107, 108].

Abnormal activation of Wnt, Notch, Hh, and Hippo pathways has been shown to facilitate the maintenance of CSC properties and promote tumor progression. Moreover, these pathways interact with other oncogenic cascades, such as RAF/MEK/ERK, PI3K/Akt, TGF-β, EGFR, STAT3, and NF-κB to further enhance the tumorigenicity of CSCs. Therefore, targeting the WNT, Notch, Hh, and Hippo pathways and related oncogenic pathways is crucial for the eradication of CSCs and the prevention of tumor progression.

### Biomarker- and pathway-targeting strategies

#### Biomarker-targeting strategies

Selective targeting of CSCs is an effective strategy to inhibit cancer progression and reduce risk of tumor relapse. Clinical trials have shown that suppressing the expression of CSCs biomarkers can reduce CSCs stemness [109–118] (Table 1). The interaction between CD44 and HA is associated with poor prognosis, and CD44 has been shown to be an important biomarker for CSCs. RG7356, for example, is a recombinant anti-CD44 immunoglobulin G1 humanized monoclonal antibody. It binds specifically to the HA-binding region of the extracellular domain of all CD44 heterodimers, to inhibit the interaction between HA and CD44, and has exhibited growth inhibition effect on several tumors in vitro [109]. A randomized phase II trial of HA-irinotecan on CD44-expressing SCLC showed significant clinical benefit in patients, suggesting that delivery of chemotherapeutic agents to activated CD44, thereby abrogating the interaction of CD44 with HA, is a compelling approach to cancer treatment [110]. CD133 has been reported to play a role in tumor spread and is considered a good candidate for targeting CSCs. A phase II clinical study provides preliminary evidence that CART-133 cells have antitumor activity with a manageable safety profile in advanced HCC [118]. High expression of EpCAM has been observed on CSCs and targeting EpCAM is an effective strategy for cancer treatment. A phase I clinical trial indicated that VB4-845 successful blocks tumor growth in patients with high EpCAM expressing non-muscle-invasive bladder cancer [115]. However, although in vitro experiments have shown significant anticancer effects, clinical trials of adecatumumab, huKS-IL-2, and catumaxomab targeting EpCAM exhibited limited clinical benefits for cancer patients [114, 116, 117]. ALDHs have been evaluated as potential prognostic markers of cancer. In a clinical trial of curcumin and curcumin combined with 5-fluorouracil/oxaliplatin in patients with colorectal liver metastases (CRLM), curcumin alone and in combination significantly reduced the number of spheroids and ALDH-active cells. Curcumin enhanced anti-proliferation and apoptosis effects of 5-fluorouracil/oxaliplatin and reduced the expression of stem cell associated markers ALDH and CD133 [111]. Nevertheless, no clinical benefits were observed in phase II trials of paclitaxel (in combination with reparixin) and disulfiram [112, 113]. In summary, targeting CSC biomarkers to eradicate CSCs selectively is expected to be an effective strategy to inhibit cancer progression and reduce the risk of tumor relapse.

**Table 1:** Clinical trials of different strategies for eradicating CSCs

Therapies	Agents	Targets	Diseases	Clinical trial ID	Study phase	Ref.
Biomarker-targeting strategy	RG7356	CD44	Solid tumors	NCT01358903	Phase I	[109]
	HA-irinotecan	CD44	Extensive-stage SCLC	/	Phase IIa	[110]
	CART-CD133	CD133	HCC	NCT02541370	phase II	[118]
	Curcumin	ALDH	CRC	NCT01490996	Phase I	[111]
	Paclitaxel (with reparixin)	ALDH	TNBC	NCT01861054	Phase II	[112]
	Disulfiram	ALDH	Germ cell tumor	NCT03950830	Phase II	[113]
	Adecatumumab	EpCAM	Hormone refractory prostate cancer	/	Phase I	[114]
	VB4-845	EpCAM	Nonmuscle-invasive bladder cancer	/	Phase I	[115]
	huKS-IL2	EpCAM	Advanced solid tumors	NCT00132522	Phase Ib	[116]
	Catumaxomab	EpCAM	Epithelial cancer	NCT01320020	Phase I	[117]
Pathway-targeting strategy	WNT974	Porcupine	CRC	NCT01351103	Phase I	[119]
	Ipafricept (IPA) with nabpaclitaxel/ gemcitabine	FZD	Pancreatic cancer	NCT01351103	Phase Ib	[120]
	Vantictumab (OMP‑18R5) with paclitaxel	FZD	HER2-nagetive BC	NCT01973309	Phase Ib	[121]
	E7449	Tankyrase	Advanced solid tumor	NCT01618136	Phase I	[122]
	CWP232291	β-catenin complex	AML	NCT01398462	Phase I	[123]
	Acylhydrazones	β-catenin complex	AML	NCT00990587	Phase I	[124]
	Cirmtuzumab	ROR1	Chronic lymphocytic leukemia	NCT02222688	Phase I	[125]
	Collagenase (CHH)	YAP	Uterine fibroids	NCT02889848	Phase I	[126]
	PF-03084014	GSIs	Desmoid fibromatosis	NCT00878189	Phase I	[127]
	OMP-59R5	GSIs	Solid tumors	NCT01277146	Phase I	[128]
	BMS-986115	GSIs	Advanced solid tumor	NCT01986218	Phase I	[129]
	RO2929097	GSIs	High grade gliomas	NCT01119599	Phase 0/I	[130]
	MK-0752	GSIs	Pancreatic ductal adenocarcinoma	NCT01098344	Phase I	[131]
	RO4929097 (with Vismodegib)	GSIs	Advanced sarcoma	NCT01154452	Phase Ib/II	[132]
	LY900009	GSIs	Advanced-stage cancer	NCT01158404	Phase I	[133]
	Demcizumab (with pemetrexed/ carboplatin)	Notch ligand	NSCLC	NCT01189968	Phase IB	[134]
	Rovalpituzumab tesirine	Notch ligand	Extensive-stage-SCLC	NCT03033511	Phase III	[135]
	Enoticumab	Notch ligand	Advanced-stage solid tumors	NCT00871559	Phase I	[136]
	Brontictuzumab	Notch receptor	Solid tumors	NCT01778439	Phase I	[137]
	Tarextumab (with etoposide/cisplatin)	Notch receptor	Extensive-stage SCLC	NCT01859741	Phase II	[138]
	Vismodegib (GDC-0449)	SMO	Advanced Basal-Cell Carcinoma	NCT00607724	Phase I	[139]
	IPI-926	SMO	Solid tumors	/	Phase I	[140]
	TAK-441	SMO	Advanced solid tumors	NCT01204073	Phase I	[141]
	PF-04449913	SMO	Advanced solid tumors	NCT01286467	Phase I	[142]
	LDE225	SMO	Extensive stage SCLC	01579929	Phase I	[143]
	LY2940680	SMO	Advanced/metastatic cancer	NCT01226485	Phase I	[144]
	Sarigegib (with cetuximab)	GLI	Recurrent/metastatic head and neck squamous cell carcinoma	NCT01255800	Phase I	[145]
	Itraconazole	GLI	Biochemically relapsed prostate cancer	NCT01787331	Phase II	[146]
	AZD9150	STAT3	Lymphoma/lung cancer	/	Phase I	[147]
	OPB-111077	STAT3	Advanced Cancers	NCT01711034	Phase I	[148]
	Alpelisib	PI3K	Epithelial ovarian cancer	NCT01623349	Phase Ib	[149]
	Duvelisib	PI3K	T-cell lymphoma	NCT01476657	Phase I	[150]
TME-targeting strategy	Bintrafusp Alfa	TGF-β/ PD-L	NSCLC	NCT02517398	Phase I	[151]
	M7824	TGF-β	Advanced solid tumors	NCT02517398	Phase I	[152]
	Galunisertib	TGF-β	Advanced rectal cancer	NCT02688712	Phase II	[153]
	FIGHT-101	FGF/ FGFR	Advanced malignancies	NCT02393248	Phase I/II	[154]
	Fruquintinib	VEGF/ VEGFR	metastasis CRC	NCT02314819	Phase III	[155]
	Ramucirumab /Pembrolizumab versus Standard of care (SOC)	VEGF/ VEGFR	NSCLC	NCT03971474	phase II	[156]
	Rilotumumab (AMG 102)	HGF	NSCLC	NCT02318368	Phase I/II	[157]
	Siltuximab	IL-6	Advanced solid tumors	/	Phase I/II	[158]
Immune-targeting strategy	Neo-DCVac	DC	NSCLC	NCT02956551	Phase I	[159]
	DCs in combination of NAC-AC	DC	Breast cancer	NCT03450044	Phase I/II	[160]
	DCs in combination of poly-ICLC	DC	Pancreatic cancer	NCT01410968	Phase I	[161]
	DCVax-L plus SOC	DC	Glioblastoma	NCT00045968	Phase III	[162]
	Decitabine (with talazoparib)	DNMT	AML	NCT02878785	Phase I	[163]
	Azacitidine (with pembrolizumab)	DNMT	Colorectal cancer	NCT02260440	Phase II	[164]
	Guadecitabine (with GM-CSF)	DNMT	Advanced colorectal cancer	NCT01966089	Phase I	[165]
	Abexinostat	HDAC	Solid tumor malignancies	EudraCT-2009–013691-47	phase II	[166]
	Panobinostat	HDAC	AML	/	Phase I	[167]
	Entinostat	HDAC	HER2 + metastatic BC	NCT02833155	Phase I	[168]
	Tazemetostat	EZH2	B-cell non-Hodgkin lymphoma	/	Phase II	[169]
	GSK2816126	EZH2	solid tumors or B-cell lymphomas	NCT02082977	Phase I	[170]
	SHR2554	EZH2	Mature lymphoid neoplasms	NCT03603951	Phase I	[171]
	Nivolumab (with chemotherapy)	PD-1	Oesophageal adenocarcinoma	NCT02872116	Phase III	[172]
	Pembrolizumab	PD-1	Advanced Colorectal Cancer	NCT02563002	Phase III	[173]
	Balstilimab/Zalifrelimab	PD-1/ CTLA-4	Advanced cervical cancer	NCT03495882	Phase II	[174]
	Tremelimumab	CTLA-4	HCC	NCT01853618	Phase I/II	[175]
	LY3415244	PD-L1/ TIM-3	Advanced solid tumors	NCT03752177	Phase I	[176]
	Atezolizumab	PD-L1	Non-squamous NSCLC	JapicCTI-184038	Phase II	[177]
	Pembrolizumab	PD-L1	NSCLC	NCT02142738	Phase III	[178]
	Tiragolumab	PD-L1	NSCLC	NCT03563716	Phase III	[179]
Induced differentiation strategy	ATRA (with apatinib)	Differentiation	Head and neck adenoid cystic carcinoma	NCT02775370	phase II	[180]
	ATRA	Differentiation	Adenoid cystic carcinoma	/	phase II	[181]
	ATRA (with belinostat)	Differentiation	Pancreatic cancer	NCT03307148	Phase I	[182]
	ATRA (with paclitaxel and interferon α2b)	Differentiation	Cervical cancer	/	phase II	[183]
	IDH1-vac	IDH1	Newly diagnosed glioma	NCT02454634	phase I	[184]
	Olutasidenib (FT-2102)	IDH1	Relapsed or refractory IDH1-mutant glioma	NCT03684811	Phase Ib/II	[185]
	Ivosidenib	IDH1	Chemotherapy-refractory cholangiocarcinoma	NCT02989857	phase III	[186]
	Vorasidenib	IDH1/2	Recurrent or progressive glioma	NCT02481154	Phase I	[187]
	Temozolomide	IDH1/2	1p/19q non-co-deleted anaplastic glioma	NCT00626990	phase III	[188]
	Olaparib	IDH1/2	IDH1/IDH2-Mutant Mesenchymal Sarcomas	NCT02576444	phase II	[189]

#### Pathway-targeting strategies

**Fig. 3:**
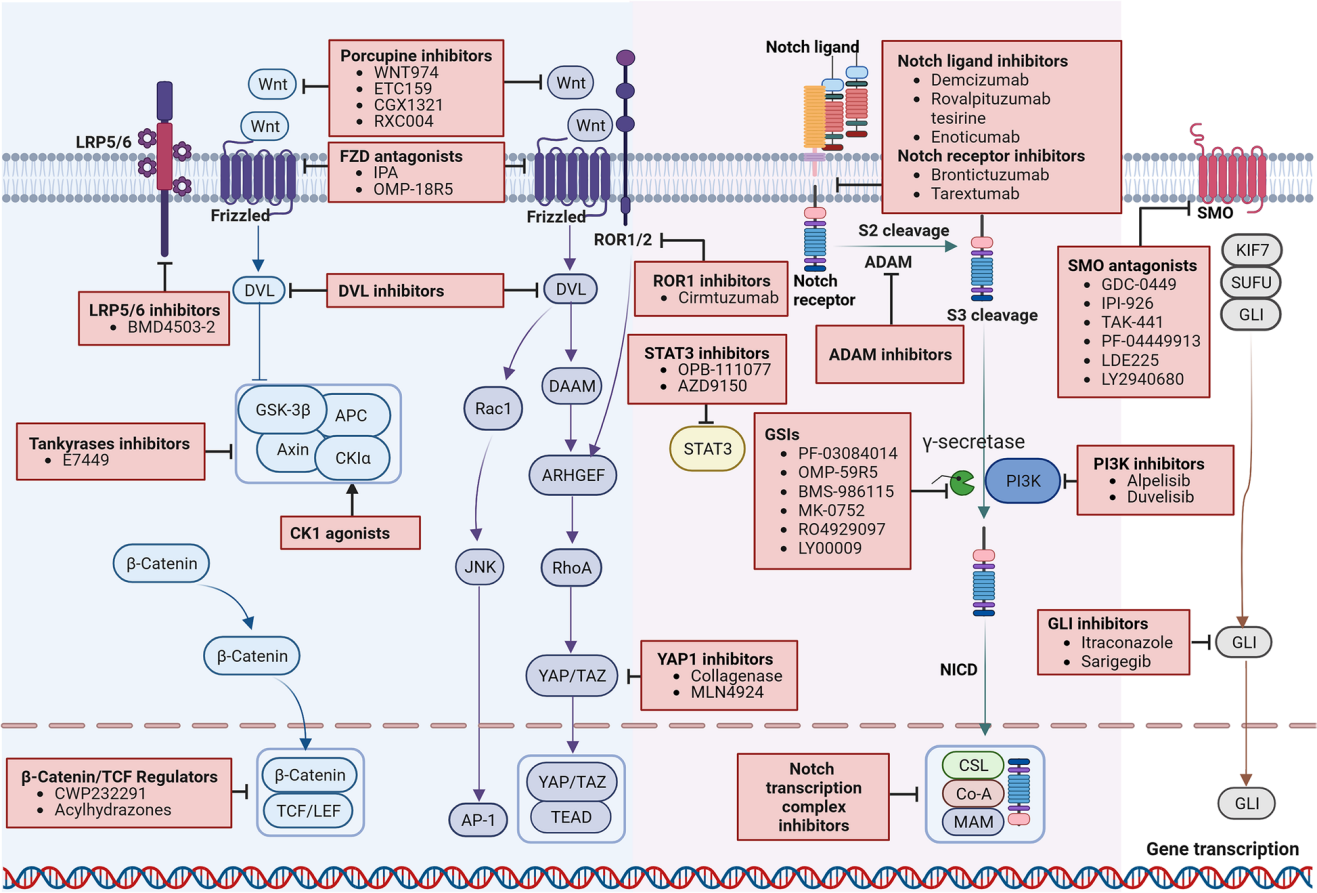
Illustration of abnormal pathways and potential targets in CSCs. FZD antagonists target either the Wnt proteins or FZD receptor complexes to inhibit the ligand-receptor interactions in both canonical and non-canonical Wnt pathway. DVL inhibitors block the DVL-PDZ interaction, resulting in subsequently inhibition of the signal transduction pathway. Tankyrase inhibitors stabilize Axin via inhibition of its proteasomal degradation, conversely resulting in increased degradation of β-catenin. CK1 agonists selectively potentiate CK1 kinase activity and stabilize the β-catenin destruction complex that decreasing Wnt signaling. β-catenin/TCF regulators inhibit Wnt-mediated transcriptional activity. LRP5/6 inhibitors competitively bind to the LRP5/6-sclerostin complex thus reverse the activation of Wnt/β-catenin signaling. ROR1-inhibitors ameliorate the access activated Wnt-signaling-mediated cancer cell proliferation, invasion, and therapy resistance. Potential anticancer therapeutic agents targeting the Notch pathway include targeting Notch ligands or receptors, inhibitors of the γ-secretase complex, and inhibitors of NICD-interacting transcriptional complex. SMO inhibitors block the Hh signaling by cyclopamine-competitively binding to SMO. GLI inhibitors prevent the transportation of GLI protein to nucleus thus decreased tumorigenesis gene expression. Inhibition of STAT3 and PI3K blocks their interactions with self-renewal pathways to facilitate CSCs eradication

Developmental signaling pathways that regulate the maintenance and survival of CSCs are potential targets to eradicate CSCs, such as Wnt, Notch, Hh, and Hippo (Table 1, Fig. 3).

Inhibition of Wnt signaling involves several critical steps, including blocking the secretion of Wnt ligands, interference with ligand-receptor binding, and modulation of intracellular signal transduction. Targeting these steps is crucial to inhibit Wnt signaling and its effects on CSCs. Porcupine inhibitors, which block the secretion of Wnt ligands, hold promise as ideal drugs for eliminating proliferative CSCs induced by canonical Wnt signaling and dormant CSCs induced by non-canonical Wnt signaling. Clinically, porcupine inhibitors have shown their therapeutic potential in various tumors. Currently, only 4 molecules, namely LGK974, ETC159, CGX1321, and RXC004, are undergoing Phase I clinical trials [119, 190]. However, the clinical use of these inhibitors may have a relatively narrow therapeutic window due to the involvement of the Wnt signaling cascade in the homeostasis of key organs and tissue [191]. Interfering with the binding of Wnt ligands to their receptors is another effective strategy to inhibit Wnt signaling. Wnt/FZD antagonists such as ipafricept (IPA; OMP54F28) and vantictumab (OMP-18R5) can directly bind to Wnt ligands or FZD receptors, competing with Wnt ligands for binding to FZD receptors [120, 121]. This competition inhibits Wnt regulatory processes in both the canonical and non-canonical Wnt pathways [192, 193]. Alternatively, blocking intracellular signal transduction may help to inhibit Wnt signaling. DVL inhibitors disrupt the interaction between DVL and PDZ, resulting in the subsequent inhibition of the signaling pathway [194, 195]. In canonical Wnt signaling, the LRP5/6 inhibitor BMD4503-2 competitively binds to the LRP5/6-sclerostin complex, thereby reversing Wnt/β-catenin pathway activation [196]. Stabilization of the β-catenin structural complex prevents the localization of β-catenin in the nucleus, making it an attractive therapeutic target. Tankyrases inhibitor, E7449, regulates the stability of AXIN by directing its ubiquitylation and proteasomal degradation, thereby increasing the activity of the destruction complex and reducing free β-catenin [122, 197]. CK1 agonists selectively potentiate CK1 kinase activity and stabilize the β-catenin destruction complex that decreasing Wnt signaling [198]. Finally, targeting the downstream effectors, such as β-catenin/TCF and β-catenin-dependent transcriptional activators, is another feasible strategy to inhibit Wnt-mediated transcriptional activity [199, 200]. Previous studies have suggested that compounds like CWP232291 and acylhydrazones block or disrupt the interaction between β-catenin and the TCF complex, suppressing Wnt target genes [123, 124]. In non-canonical Wnt signaling, ROR1-inhibitors ameliorate the access of a ctivated Wnt-signaling-mediated cancer cell proliferation, invasion, and therapy resistance [201]. Cirmtuzumab has shown obvious inhibitory effects on ROR1 expression and tumor progression in chronic lymphocytic leukemia patients [125].

Various potential anticancer therapeutics targeting the Notch pathway have been explored to eliminate CSCs including inhibiting the release of Notch ligands, blocking the proteolytic cleavage of Notch receptors, interrupting the Notch signaling transduction and inhibiting the expression of target genes associated with Notch signaling. Targeting Notch ligands or receptors can inhibit aberrant signaling initiation and decrease tumorigenesis in CSCs [135–138, 202]. Phase I Clinical trials have demonstrated the antitumor activity of DLL4-targeted agents, such as demcizumab and enotizumab, either as monotherapy or in combination therapy [134, 136]. Brontictuzumab, a Notch 1 inhibitor, has shown efficacy signal in patients with Notch 1 activation and combination therapy with other anticancer agents has improved clinical benefits [137]. However, Notch 2/3 receptor inhibitor tarextumab had limited additional effects when combined with gemcitabine and nab-paclitaxel in untreated advanced pancreatic adenocarcinoma [138]. ADAMs-catalyzed S2 cleavage occurs in the ligand-receptor binding domain, which mediates the release of the ectodermal structural domain and regulates the rate of Notch signaling [203]. Notch signaling from extracellular to intracellular relies heavily on the γ-secretase complex-mediated final cleavage. γ-secretase inhibitors (GSIs) block the S3 cleavage of Notch receptors, preventing the release of NICO and subsequent activation of Notch signaling [204]. Several GSIs including PF-03084014, OMP-59R5, BMS-986115, RO2929097, MK-0752, RO4929097, and LY900009, have been investigated in clinical trials [127–133]. However, dose-limiting toxicities have been observed with most GSIs. A potentially superior option to GSIs is the use of γ-secretase modulators (GSMs). GSMs modify the catalytic activity of γ-secretase rather than non-selectively inhibiting it, thereby preserving some Notch signaling function and theoretically reducing side effects [205]. Activating target gene transcription is the final step in Notch signaling transduction. Disrupting the Notch transcriptional complex downstream of abnormal Notch activation has advantages in repressing the expression of Notch targeted genes [206]. Although inhibition of the Notch pathway has shown significant anti-tumor efficacy in preclinical research, these results have not been consistently identified in clinical trials.

Inhibiting the expression of Hh ligands, SMO and GLI transcription factors is thought to be an effective way to suppress the over activation of the Hh signaling in CSCs. Clinical trials are currently underway for agents targeting these components [207]. Intracellular cyclopamine is known to inhibit the activity of the Hh pathway through directly binding and inactivating SMO. Cyclopamine derivatives also show SMO inhibitory effect by competitively binding to SMO, thereby blocking Hh signaling [208]. Several SMO inhibitors, including GDC-0449, IPI-926, TAK-441, PF-04449913, LDE225, and LY2940690 were under clinical trials, all of which showed anticancer effects through interrupting Hh signaling [139–144]. However, tumor cells frequently acquire resistance to SMO inhibitors through SMO mutation [209]. Furthermore, SMO antagonists have limited efficacy when aberrant Hh activation occurs due to genetic alterations downstream of SMO, or SMO-independent activation of GLI transcription factors. In this context, the development of GLI inhibitors is considered an alternative strategy [210]. In preclinical studies, GLI inhibitors have shown promising results. Encouraging anticancer effects was observed in Sarigerib treated recurrent/metastatic head and neck squamous cell carcinoma patients. Mechanical research indicated that the clinical response was associated with the regulation of Hh signaling pathway [145]. Importantly, targeting both SMO and GLI, in a synergistic inhibition of the Hh pathway, provides stronger efficacy than monotherapy [211]. Endoplasmic reticulum aminopeptidase 1 (ERAP1) plays a positive role in regulating Hh signaling. Inhibition of ERAP1 controls Hh-induced tumor growth, suggesting that ERAP1 is a promising therapeutic target in Hh over-activated tumors [212]. Continued research and clinical trials are necessary to further explore and optimize strategies targeting the above signaling pathway for the effective treatment of CSCs and related cancers.

Inhibition of YAP/TAZ activity is a potential strategy to eliminate CSCs, while the direct inhibitors of YAP/TAZ are still under development [86]. A clinical trial of collagenase on patients with uterine fibroids showed that collagenase decreased the expression of cell proliferation marker and phosphorylated YAP [126]. Inhibition of upstream Hippo kinases can be an attractive strategy to suppress tumor progression. Loss of LATS1/2 in tumor cells inhibits tumor growth and enhances anti-tumor immune response [213].

On the other hand, interfering the interaction between pathways might help to eradicate CSCs. STAT3 inhibitor, AZD9150, has showed preclinical activity in patients with highly treatment-refractory lymphoma and NSCLC in a phase I dose-escalation study [147]. OPB-111077, a novel inhibitor of STAT3 and mitochondrial oxidative phosphorylation, showed notable clinical activity in a subject with diffuse large B-cell lymphoma [148]. PI3K inhibitor, alpelisib, provided preliminary clinical activity in epithelial ovarian cancer [149]. Duvelisib (IPI-145) is an oral inhibitor, which demonstrated promising clinical activity and an acceptable safety profile in relapsed/refractory T-cell lymphoma [150].

## TME and TME-targeting strategies

**Fig. 4:**
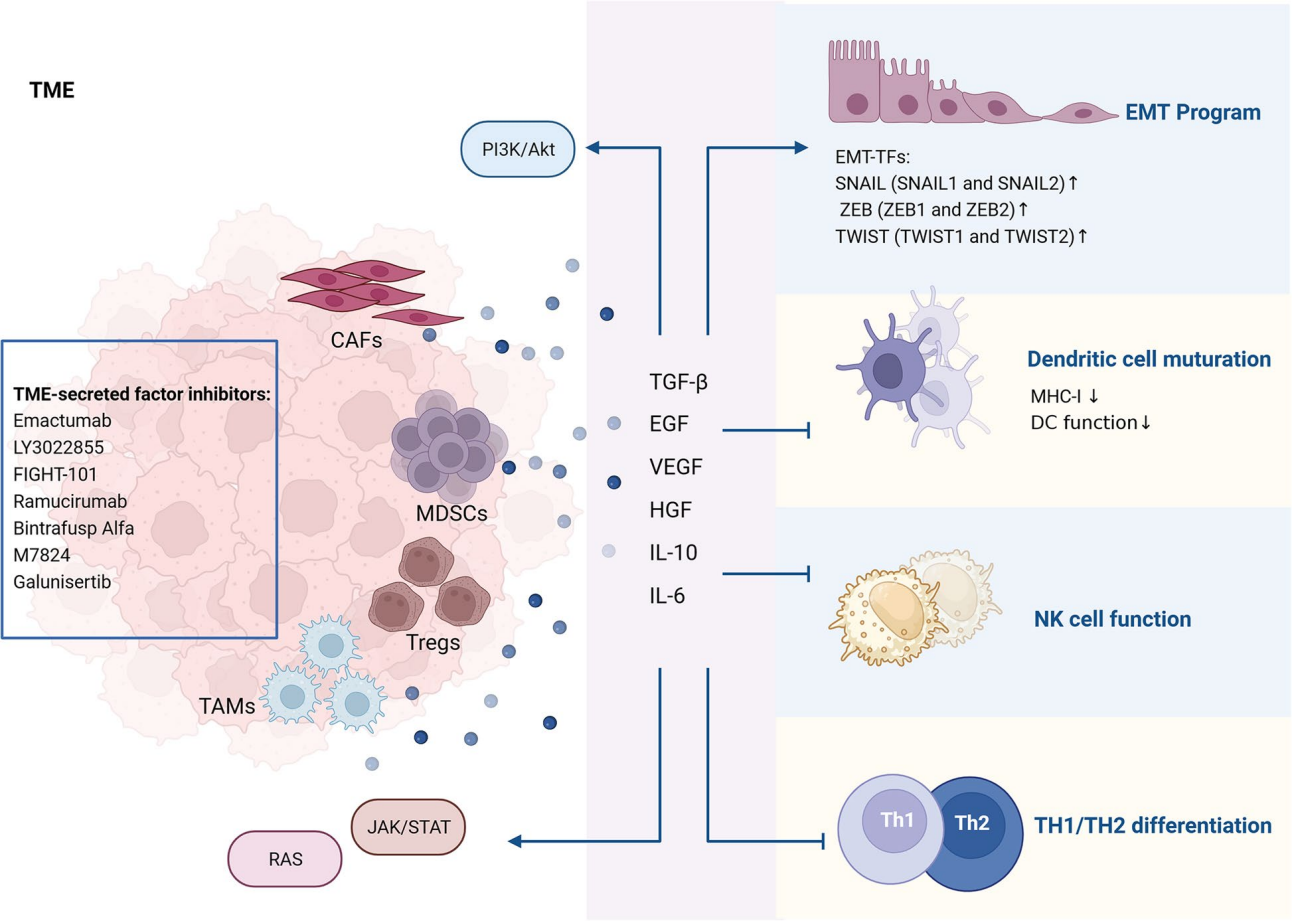
TME-induced immunosuppressive environment for the maintenance of CSC properties. Cytokines and growth factors from CAFs, Tregs, MDSCs and TAMs activate cancer-related EMT and suppress DC cell maturation, NK cell function, and TH1/TH2 differentiation. Additionally, TME-derived factors activate cancer-associated pathways including PI3K/Akt, JAK/STAT, and RAS

The TME play a crucial role in maintaining CSC properties and creating an inflammatory and immunosuppressive niche [214]. TME-induced chronic inflammation and immunosuppressive niche are closely related to the high cancer incidence. A prolonged and unresolved inflammatory response leads to the aberrant activation and accumulation of various stromal cells, which disrupts the normal function of stromal cells in maintaining homeostasis and promotes EMT activation and the formation of a tumorigenic environment. Furthermore, exosomes secreted from CSCs and stromal cells contribute to drug resistance and tumor immunosuppression environment formation, thus promoting CSC stemness [215, 216]. A properly functioning host immune system is essential to prevent inflammation-induced dysregulation of TME homeostasis. However, the recruitment and activation of stromal cells can suppress the host immune system [217]. On this basis, various strategies targeting EMT have been employed to eliminate CSCs (Table 1, Fig. 4).

### The role of TME in CSC formation

Cancer associated fibroblasts (CAFs) within the TME play an integral role in maintaining CSCs and promoting drug resistance. Therapeutic agents have been developed to directly target surface markers on CAFs, such as PAF, S1004A, and TEM8, in order to control tumor growth [218]. Moreover, cytokines and growth factors secreted by CAFs, including TGF-β, IL-6, EGF, VEGF, and HGF, have been reported to facilitate tumor progression [219]. Within the TME, several types of immune cells contribute to chronic inflammation and promote an immunosuppressive environment. These include regulatory T cells (Tregs), myeloid-derived suppressor cells (MDSCs), and inflammatory tumor-associated macrophages (TAMs). Tregs are a subset of CD4^+^ T cells characterized by the expression of FoxP3 in the nucleus and CD25 and CTLA-4 on the cell surface. They exert significant immunosuppressive effects by producing immune inhibitory cytokines such as TGF-β and IL-10 as well as expressing immune checkpoint molecules like PD-1, CTLA4, LAG3, TIM3, and TIGIT [220]. TAMs are one of the most abundant infiltrating immune cells in TME [221]. Immunosuppressive factors from TAMs, such as TGF-β and IL-10, can stimulate the activity of Tregs and suppress the immune activity of T-cells, leading to immune escape. TAMs also secreted pro-inflammatory cytokines, such as IL-6, which activate signaling pathways like STAT3, PI3K/Akt, cyclooxygenase 2 (COX2), prostaglandin E2 (PGE2), β-catenin, and Ras-MARK pathways. Activating of these pathways alters the expression patterns of genes associated with proliferation, survival, and cell cycle regulation, thereby promoting cancer cell invasion and drug resistance [222, 223]. MDSCs support tumor progression through promoting cancer cell survival, angiogenesis, invasion, and metastasis. Expansion and recruitment of MDSCs driven by TME and their secreted inflammatory factors (e.g., TGF-β, VEGF, IL-10, IL-12, IL-13, etc.) mediate chronic inflammatory and immunosuppressive activity [224]. Elevated TGF-β has been proved to suppress the effector function of nature killer (NK) cell, hinder dendritic cell (DCs) function, and prevent TH1 and TH2 cell differentiation, while promoting TH17 and Treg cell programme [225]. Modulation of CAFs, Tregs, TAMs, and MDSCs may alleviate immunosuppressive and inflammatory TME, thus increase sensitivity of CSCs to treatment.

### TME-targeting strategies

Regulation of the secreted growth factors and cytokines is one of the main strategies to modulate TME. Among these growth factors, TGF-β is one of the most extensively studied cytokines derived from TME. TGF-β signaling in stromal cells contributes to cancer progression by suppressing T-cell responses and regulating immune escape [225]. In addition, Non-canonical TGF-β has been reported to activate non-Smad pathways, including MAPK, YAP/TAZ, PI3K/Akt, and AMPK signaling, leading to fibrosis, immune evasion, and EMT, ultimately promoting cancer progression [226]. At present, TGF-β-targeting agents have been designed and have achieved satisfactory clinical activity. Clinical trials of M7824 and galunisertib in cancer patients have shown encouraging clinical efficacy by targeting TGF-β [152, 153]. EGF/EGFR interact with the MEK-ERK and AKT-PI3K signaling pathways, which leads to cancer cell proliferation [227]. Afatinib or olmutinib in combination with conventional chemotherapeutic agents improve CSC eradicating efficacy by inhibiting EGFR tyrosine kinase and ATP-binding cassette subfamily G member 2 (ABCG2) [228, 229]. VEGF/VEGFR interaction involves the activation of downstream pathways, including Ras-Raf-MAPK, AKT-mTOR, and Scr-FAK. Activation of these pathways promotes cell survival, proliferation, migration, and differentiation [227]. A randomized clinical trial indicated that fruquintinib, a VEGFR inhibitor, increased overall survival (OS) in patients with metastatic CRC [155]. Ramucirumab increased the sensitivity of cancer cells to immune checkpoint inhibitors (ICIs) and improved OS by inhibiting VEGF/VEGFR [156]. HGF from TME has been shown to regulate the innate resistance of BRAF-mutant cancer cells to RAF inhibitors by activating the MAPK and PI3K/Akt signaling pathways in cancer cells [230]. A phase I/II clinical trial evaluating rilotumumab, an anti-HGF antibody, in combination with erlotinib in patients with metastatic NSCLC showed that the combination of rilotumumab and erlotinib was more effective than erlotinib alone [157]. Suppressing TME-induced cytokines production can help restore antitumor immunity and sensitize tumors to immunotherapy. IL-6 plays a critical role in inflammation and cancer development. It has been reported that high levels of IL-6 confer resistance to cisplatin in patients with non-small-cell lung cancer (NSCLC) [231]. However, siltuximab, an anti-IL-6 monoclonal antibody, showed limited efficacy in patients with advanced solid tumors [158].

## CSCs and immunoevasion

**Fig. 5:**
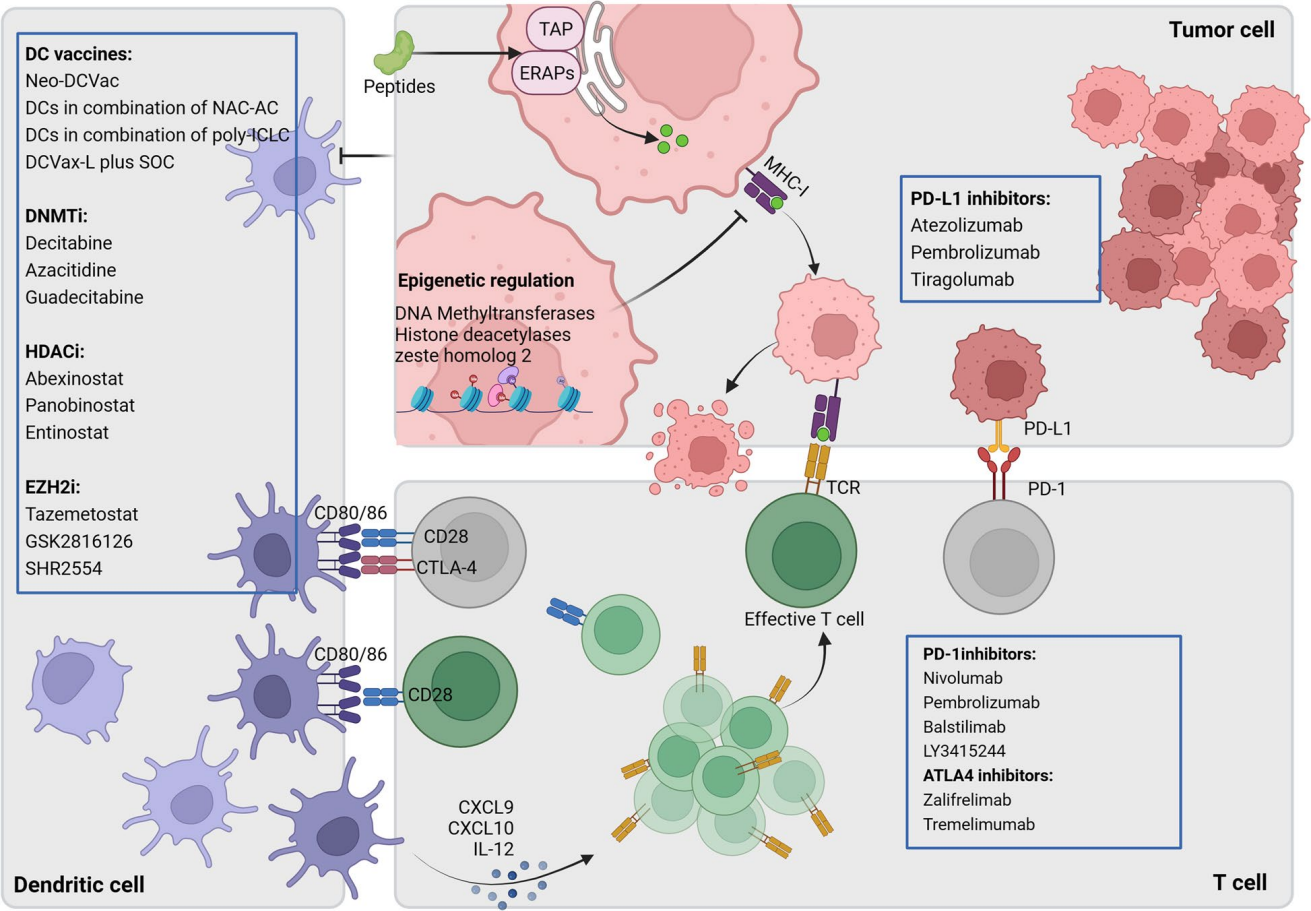
DC and T cell mediated tumor immunology and immunotherapy. In MHC-I antigen presentation pathway, oligopeptides degraded from cytosolic and nuclear protein are taken up and translocated into ER by TAP and further trimmed by ERAPs. The modified peptides bind to MHC-I and are transported to the cell surface for exposure to CD8 + T cells [233]. DC produce CXCL9, CXCL10 and IL-2 to recruit effective T cells therefore increase immune response. Activation of CTLA-4 and PD1/PDL1 reprogrammed immune homeostasis and induced cancer cells to eliminate T cell function. Immunotherapies that inhibit CTLA-4 and PD1/PDL1 and enhance MHC-I expression can effectively modulate DC function and improve cancer immunotherapy

Tumor immunosurveillance is orchestrated by tumor immunogenicity and immunoevasion, immune cell infiltration, and T cell checkpoints [232]. Stromal cells and recruited immune cells in TME collectively formed an immunosuppressive niche that facilitate﻿s the maintenance and proliferation of CSCs. Furthermore, dysregulated cellular antigen processing and presentation machinery, as well as upregulated expression of immune checkpoint molecules allow CSCs to escape from immune surveillance (Fig. 5).

CD8^+^ T lymphocytes play an indispensable role in regulating adaptive immune responses. They detect antigenic peptides bound to MHC-I perceptively and efficiently eliminate abnormal cells, thereby preventing cancer cell colonization. Stimulation with interferon-gamma (IFN-γ) can upregulate the expression of MHC-I antigen presentation components [233]. While these compositions (TAP, ERAPs, IFN-γ) are not strictly required for cell proliferation, their loss results in a reduction of pathway function and decreased cell surface levels of MHC-I molecules [234]. The presentation of cancer-associated peptide antigen by MHC-I is an important step for antitumor CD8 + T cell responses. However, CSCs have mapped out strategies to reduce antigen presentation thus to escape immune recognition, including inhibition of DC function and downregulation of MHC-I expression [235]. A growing number of studies demonstrated that CSCs alter DC phenotypes and impair their recruitment to limit them to activate T cells [236, 237]. On the other hand, activation of immune checkpoint pathways in the TME, including CTLA-4 and PD1/PDL1, reprograms immune homeostasis and enables cancer cells to evade immune attack [238]. ICIs that block CTLA-4 and PD1/PDL1 have demonstrated promising therapeutic efficacy in various human cancers. However, the effective antitumor responses of ICIs often require the secretion of IL-12 by DCs [239]. Additionally, ICIs induce, instead of relieve, T cell dysfunction in situation of low MHC-I levels [240]. In this basis, enhancement of antigen processing and presentation machinery and/or combination with ICIs may be an attractive strategy for CSCs eradication.

Type 1 conventional DCs (cDC1s) are a major subset of DCs that respond to invasive pathogens and antitumor immunity via antigen presentation to cytotoxic CD8^+^ T cells [241]. The cDC1s facilitate the differentiation and recruitment of tissue-resident memory CD8^+^ T cells within the TME by producing CXCL9 and CXCL10 to activate STING pathway. In addition, cDC1s-derived IL-12 increases the sensitivity of cancer cells to ICIs by augmenting CD8^+^ T cell activation. Adequate CD8^+^ T cell activation, in turn, induces the maturation and migration of cDC1s to the draining lymph nodes [235]. Therefore, DCs serve as a potent tool for motivating antitumor responses to eradicate CSCs effectively. DC vaccines are currently under clinical investigation and have shown promising therapeutic modality. DC vaccines, including Neo-DCVac, autologous DCs in combination with doxorubicin and cyclophosphamide (NAC-AC) or toll-like receptor agonist have demonstrated efficacy in restraining tumor progression by promoting T cell mediated immunity and sensitizing cancer cells to ICIs (Table 1) [159–162].

The binding of MHC-I with specific peptides and their presentation on the cell surface is an indispensable step in antitumor immunity. Loss expression of MHC-I renders CSCs invisible to the immune system. Clinical evidence has confirmed that advanced melanoma patients with low levels of MHC-I on the cancer cell surface derived limited benefit from ICIs therapy [242]. The expression level of MHC-I is dependent on NOD-like receptor family CARD domain containing 5 (NLRC5), which is regulated by IFNγ-activated STAT1 signaling [243]. Yet no clinical trial records regarding the regulation of MHC-I by NLRC5 in the PubMed database. The downregulated expression of MHC-I is associated with repressive histone modifications of Lys-27 in histone 3 (H3K27m3), including hypermethylation, histone deacetylation and trimethylation. These modifications are partly regulated by enhancer of zeste homolog 2 (EZH2). Accordingly, inhibition of DNA Methyltransferases (DNMTi), Histone deacetylases (HDACi), and EZH2 (EZH2i) theoretically has the potential to increase the expression of MHC-I [244]. The efficacy of these drugs, either as monotherapy or in combination with other treatments, has been evaluated in clinical trials. Some of these trials have demonstrated improved sensitivity of cancer cells to drug administration (Table 1) [163–169]. For instance, SHR2554, an EZH2 inhibitor, has shown promising antitumor activity in patients with relapsed or refractory follicular lymphoma, peripheral T-cell lymphoma, and classical Hodgkin lymphoma [171]. EZH2 inhibitor tazemetostat showed encouraging efficacy in patients with R/R EZH2 mutation-positive follicular lymphoma with a manageable safety profile in the overall population [169]. HDAC inhibitors including abexinostat, panobinostat, and entinostat have shown antitumor efficacy in clinical trials [166–168]. However, DNMT inhibitors, such as decitabine, azacitidine, and guadecitabine, have shown limited clinical activities on tested cancers [163–165].

T cells are activated through a complex interplay involving antigen specific T cell receptor recognition of peptides presented by MHC molecules and interactions between membrane proteins on antigen-presenting cells (APCs, including CD80 and CD86) and CD28 on T cells [245]. CTLA-4 inhibits the activation of T cells by competing with CD28 for binding to CD80 and CD86 on APCs, thereby attenuating CD8^+^ T cell responses. Another important immune checkpoint pathway involves the PD-1 and its ligand PD-L1. The expression of PD-1 on T cells and PD-L1 on tumor cells or other immune cells is considered a hallmark of T cell dysfunction and promotes T cell exhaustion [246]. Blockade of the expression of CTLA-4 and the interaction between PD-1 and PD-L1, has been shown to restore T cell function and enhance antitumor responses during chronic infections and in the TME [247]. ICIs specifically target these immune checkpoint molecules and modify the immune environment, have demonstrated the effectiveness of ICIs in various cancers (Table 1) [172–179]. Nivolumab and pembrolizumab, the PD1 inhibitors, have shown longer OS/FPS in multiple cancers compared to chemotherapy in clinical trials [172, 173]. Balstilimab (PD-1 inhibitor) and zalifrelimab (CTLA-4 inhibitor) are checkpoint inhibitors emerging as promising investigational agents for the treatment of advanced cervical cancer. A phase II clinical trial indicated that the combination of balstilimab and zalifrelimab had a high proportion of complete responses and efficacy in patients with recurrent and/or metastatic cervical cancer [174]. Tremelimumab is a fully monoclonal antibody that binds to CTLA-4 on the surface of activated T cells, which triggered the accumulation of intratumoral CD8 + cells in patients with advanced HCC [175]. PD-L1 inhibitors, such as atezolizumab, pembrolizumab, tiragolumab, improve objective response rates and are associated with significantly longer PFS and OS [177–179]. However, LY3415244, a TIM-3/PD-L1 inhibitor designed to overcome primary and acquired anti-PD-(L)1 resistance, was terminated early due to unexpected immunogenicity [176].

## Agent-induced CSC differentiation

CSC-induced poorly differentiated cancers are more malignant, differentiation therapy is therefore a strategy to inhibit tumorigenesis by inducing the conversion of highly malignant undifferentiated cancer cells into benign differentiated cells. Several agents, including retinoic acid (RA), cAMP, sodium butyrate and cytokines, have been proved to induce cell differentiation in specific types of cancer. ATRA induces terminal differentiation and exhibits significant anticancer effect in patients with AML and acute promyelocytic leukemia (APL) [248]. Ongoing research is exploring the potential of ATRA and its derivatives for differentiation therapy in non-AML/APL [180, 181, 183]. New differentiation-inducing drugs that inhibit mutant isocitrate dehydrogenase (IDH) 1 and IDH2 have shown differentiating potential clinically, which have been approved for AML therapy. Mutations in IDH1 and IDH2 render their functional activity in differentiation regulation, particularly by increasing histone and DNA methylation [249]. Furthermore, epigenetic regulatory inhibitors such as DNMTi and HDACi, which promote MHC-I expression, have also demonstrated their involvement in cell differentiation. IDH1/2 mutations have been observed in solid tumors, and clinical trials are underway to evaluate the effectiveness of IDH1/2 inhibitors in inducing CSC differentiation in solid tumors. Continued research in differentiation therapy holds promise for expanding its application across various cancer types (Table 1) [184–189]. IDH1-Vaccine or IDH1-targeting agents including ivosidenib and olutasidenib showed clinical benefit in cancer patients in terms of reduced tumor burden and increased PFS [184–186]. Vorasidenib, a dual IDH1/2 inhibitor, showed preliminary antitumor efficacy in patients with recurrent or progressive nonenhancing mIDH lower grade gliomas [187].

## Other strategies of target CSCs

Cancer is a complex disease that affects the health of people around the world. Chemotherapy remains an important treatment for cancer, despite advances in surgery and radiotherapy. Current treatments are expensive and are associated with many adverse events. In addition, cancer cells become resistant to chemotherapy as treatment progresses, making it difficult for patients to benefit from unmodified chemotherapy. The development of new drugs remains a top priority, but with the financial burden of drug research, drug repurposing is an innovative way to update the chemotherapy arsenal [250]. Metformin is the first choice for treating type 2 diabetes because of its robust glucose-lowering effects, well-established safety profile and relatively low cost [251]. However, metformin is repurposed as an anti-cancer agent. A clinical trial indicated that metformin has showed anticancer efficacy by inhibiting CD133 [252]. In addition, a phase II clinical trial showed that metformin treatment resulted in a significant reduction in the CSC population and alteration of DNA methylation of chemoresistance carcinoma-associated mesenchymal stem cells (CA-MSCs), which eliminated CA-MSC–driven increases in chemoresistance [253]. The drug repurposing strategy provides an alternative for cancer treatment.

## Conclusion and future perspectives

Despite ongoing debates regarding the origin and specific characteristics of CSCs, it is widely acknowledged that these cells have exhibited stemness properties such as self-renewal, proliferation, differentiation, and therapy resistance. Therefore, targeting CSCs with different therapeutic agents holds great promise for future antitumor treatments. Currently, most cancer therapies only control the growth and proliferation of CSCs instead of completely eradicating the tumor bulk. In this context, researchers are exploring the modulation of abnormal signaling pathways, inhibition of CSC specific proteins, and regulation of the immune environment to gain new insights into cancer treatment strategies. However, several challenges remain a significant hurdle, as it is crucial to specifically target CSCs while minimizing damage to normal cells. Based on the plasticity and heterogeneity of CSCs, precision oncology will be the future trend of tumor therapy. Selecting the right combination of drugs for each patient and using them at the right stage of the disease require a comprehensive understanding of the biomarkers, stemness-associated pathways, TME, and immune mechanisms in CSCs. Additionally, efforts are ongoing to mitigate adverse effects associated with treatment, and explore innovative approaches for delivering therapeutic agents and maintaining effective drug concentrations. Continued research and development in these areas hold the potential to revolutionize cancer treatment by specifically targeting CSCs, overcoming therapy resistance, and achieving more comprehensive and durable therapeutic outcomes.

## Abbreviations

CSCs: Cancer stem cells
TME: Tumor microenvironment
AML: Acute myeloid leukemia
ELDA: Extreme Limiting Dilution Assays
EMT: Epithelial-mesenchymal transition
ALDH: Aldehyde dehydrogenase
EpCAM: Epithelial cell adhesion molecule
HA: Hyaluronic acid
ECM: Extracellular matrix
TGF-β: Transforming growth factor-beta
VEGF: Vascular endothelial growth factor
MMPs: Matrix metalloproteinase
HDAC6: Histone deacetylase 6
TCF: T-cell factors
RIP: Intramembrane proteolysis
FHL2: Four-and-a-half LIM domains protein 2
ROS: Reactive oxygen species
RA: Retinoic acid
TIF-2: Hypoxia-inducible transcription factors 2
ATRA: All-trans retinoic acid
NRF2: Nuclear factor erythroid 2-like 2
FZD: Frizzled
GSK3β: Glycogen synthase kinase 3β
DVL: Disheveled
ADAM: Disintegrin and metallo-proteinase
NICD: Notch intracellular domain
SHh: Sonic hedgehog
IHh: India hedgehog
DHh: Desert hedgehog
SMO: Smoothened
PTCH: Patch
Hip: Hedgehog interacting protein
GSIs: γ-Secretase inhibitors
GSMs: γ-Secretase modulators
ERAP1: Endoplasmic reticulum aminopeptidase 1
CAFs: Cancer associated fibroblasts
NSCLC: Non-small-cell lung cancer
ABCG2: ATP-binding cassette subfamily G member 2
TAMs: Tumor-associated macrophages
Treg cells: Regulatory T cells
NK cells: Nature killer cells
DCs: Dendritic cells
COX2: Cyclooxygenase 2
PGE2: Prostaglandin E2
G-CSF: Granulocyte colony-stimulating factor
TAP: Transporter associated with antigen processing
IFN-γ: Interferon-gamma
ICIs: Immune checkpoint inhibitors
IL-12: Interleukin-12
NLRC5: NOD-like receptor family CARD domain containing 5
H3K27m3: Histone modifications of Lys-27 in histone 3
DNMTi: Methyltransferases inhibitor
HDACi: Histone deacetylases inhibitor
EZH2i: Enhancer of zeste homolog 2 inhibitor
APL: Acute promyelocytic leukemia
IDH: Isocitrate dehydrogenase
